# Experiences of Newly Diagnosed Oral Cancer Patients during the First Wave of the COVID-19 Pandemic: A Qualitative Study from Pakistan

**DOI:** 10.3390/ijerph19148508

**Published:** 2022-07-12

**Authors:** Maria Ishaq Khattak, Saad Ishaq Khattak, Muslim Khan, Zohaib Khan, Lisa Dikomitis, Zia Ul-Haq, Norkhafizah Saddki

**Affiliations:** 1 School of Dental Sciences, Universiti Sains Malaysia, Health Campus, Kubang Kerian 16150, Kelantan, Malaysia; maria.iph@kmu.edu.pk; 2 Institute of Public Health & Social Sciences, Khyber Medical University, Peshawar 25000, Pakistan; drzia@kmu.edu.pk; 3 Sardar Begum Dental College, Gandhara University, Peshawar 25000, Pakistan; saadkhattak1996.sk@gmail.com; 4 Department of Oral & Maxillofacial Surgery, Khyber College of Dentistry, Peshawar 25000, Pakistan; muslim177@hotmail.com; 5 Office of Research Innovation & Commercialization, Khyber Medical University, Peshawar 25000, Pakistan; dr.zohaibkhan@kmu.edu.pk; 6 Kent and Medway Medical School, University of Kent and Canterbury Christ Church University, Canterbury CT2 7FS, UK; lisa.dikomitis@kmms.ac.uk; 7 Institute of Health and Wellbeing, University of Glasgow, Glasgow G12 8RZ, UK

**Keywords:** oral cancer, COVID-19, life experiences, health care, social support, Pakistan

## Abstract

The COVID-19 pandemic has resulted in the scaling back or postponement of non-emergency hospital services, including care of cancer patients. The present qualitative study explored the experiences of newly diagnosed oral cancer patients during the first wave of the COVID-19 pandemic in Pakistan. Patients who attended the Department of Maxillofacial Surgery, Khyber College of Dentistry in July 2020 were selected using a maximum variation purposive sampling method. Seventeen semi-structured interviews were conducted in Pashto, the local language of Khyber Pakhtunkhwa. All interviews were audiotaped, transcribed verbatim, and translated into English. Thematic content analysis yielded eight major themes: pain and generalised physical weakness, shock at diagnosis, psychological distress of the COVID-19 pandemic, faith and religion, double hit loss of employment, social isolation, social support from caregivers, and lack of support from health care professionals. In conclusion, the COVID-19 pandemic has a clear impact on the life experiences of newly diagnosed oral cancer patients. Distress due to delay in accessing health care and lack of support from health care providers are a matter of great concern. Appropriate interventions should be introduced to ensure psychological and social support strategies are in place for patients during interruptions of health care services.

## 1. Introduction

Oral cancer refers to malignant neoplasms affecting any region of the oral cavity, lips, salivary glands, and oropharynx [1]. Being one of the most prevalent malignancies worldwide, oral cancer is a debilitating public health problem [2]. The incidence varies widely between different regions of the world or even within the same countries from the minorities or subpopulations [3]. In 2018, the age-standardised incidence rate of oral cancer in both males and females were highest in Melanesia (21.2 per 100,000 and 12.0 per 100,000, respectively) and South-Central Asia (12.9 per 100,000 and 4.5 per 100,000, respectively) [4]. Pakistan is a low-to-middle-income country in South-Central Asia. The incidence rate of oral cancer in Pakistan is more than 10 per 100,000 population, and 8–10% of cancers diagnosed in the country were attributed to oral cancer [5]. Ranked first among males, oral cancer is the leading cause of cancer-related deaths in Pakistan [6].

The COVID-19 cases were first reported in Pakistan on 26 February 2020 and the low-level community spread started in early March 2020, forcing the government to impose a nationwide lockdown, including the suspension of elective and outpatient services at hospitals [7]. The curtailment of normal clinical activities in Pakistani hospitals to limit the spread of COVID-19 had led to disruptions in the care of cancer patients [8]. Although the long-term implications of delays and disruptions in health care services for cancer patients are yet to be seen, there is concern that patient outcomes may be negatively impacted [9].

Oral cancer treatment is often lengthy and complex [10]. Even under normal (non-pandemic) circumstances, oral cancer patients experience tremendous physical, psychological, and social impact from the disease and its treatment [11]. Delays in initiation and disruptions of treatment due to the COVID-19 pandemic may worsen not only the cancer prognosis but also the mental health of patients [12,13]. To the best of our knowledge, the experiences of newly diagnosed oral cancer patients during the COVID-19 pandemic has not been duly explored and reported. 

To address this gap, we conducted a longitudinal qualitative study with newly diagnosed oral cancer patients in the Khyber Pakhtunkhwa province of Pakistan. Experiences of oral cancer patients at diagnosis, six months after diagnosis, and one year after diagnosis were captured, and these follow-up times coincide with the first wave, second wave, and third wave of the COVID-19 pandemic in Pakistan, respectively. An in-depth insight into the lived experiences of oral cancer patients during the COVID-19 pandemic may assist in the development of effective models of care for patients. This paper reports findings from the baseline cross-sectional interviews that were conducted during the first wave of the COVID-19 pandemic.

## 2. Materials and Methods

### 2.1. Participants and Recruitment

Participants were recruited from the Khyber College of Dentistry, Department of Maxillofacial Surgery Peshawar, Khyber Pakhtunkhwa, Pakistan. Participants aged 18 years and above who were diagnosed with oral cancer in July 2020, irrespective of the stage of cancer, and had not undergone any treatment, were included in this exploratory research. Those patients who were severely ill, who had a cognitive impairment or other disease-related physical limitations, and those who were declared physically unfit to undergo an in-depth interview by the attending physician were excluded. 

To recruit a heterogeneous sample of newly diagnosed oral cancer patients, the maximum variation purposive sampling method was applied for selection of study participants. Following establishment of eligibility, 20 patients were invited to take part in this study via a telephone call by a member of the research team (MK). To achieve in-depth information about the research topic, participants with varying stages of oral cancer (early and advanced stages), age, sex, and education level were chosen. The necessary information regarding the study including the importance, objectives, and procedures were given. Of the 20 patients approached, 17 agreed to participate, and were put in touch with the interviewer (MIK) who contacted the patients soon after to schedule an interview session.

### 2.2. Data Collection

A semi-structured interviewer guide was developed by a multidisciplinary research team including researchers, subject specialists, and maxillofacial surgeons from Universiti Sains Malaysia (Malaysia) and Khyber College of Dentistry (Pakistan). The guide was created based on the review of the existing literature to direct the interviews and conversations towards the research topic [11]. The interviewer guide included the following topics: (1) making sense of the diagnosis, (2) understanding the physical experiences of oral cancer patients, (3) understanding the psychological experiences of oral cancer patients, (4) understanding the social experiences of oral cancer patients. 

All interviews were conducted in Pashto, the local language of Khyber Pakhtunkhwa. The interview was conducted by a single interviewer (MIK) who is a medical sociologist experienced in qualitative research, fluent in both Pashto and English. All 17 participants provided a written informed consent to take part in the study and for the interviews to be audio-recorded.

To minimise the burden on participants, the length of time for interviews was restricted to 30 min. Additionally, the interviews took place at a time and place convenient to the participants. To ensure confidentiality, only the participant and the interviewer (MIK) were present during the interview. To minimise the risk of exposing identifiable participant information, all participants were given unique study numbers. The interviews were discontinued wherein little or no new information were generated, indicating that we had reached data saturation. Additionally, a validated method called Comparative Method for Themes Saturation (CoMeTS) was used to help ensure data saturation had been achieved [14]. This method involved comparing all themes from all interviews with each other and reordering the sequence of interviews numerous times to help confirm saturation. The interviews were conducted according to strict COVID-19 preventive measures. To ensure trustworthiness and rigor of this qualitative research, criteria of credibility, dependability, transferability, and confirmability described by Lincoln and Guba [15] were used. 

### 2.3. Data Analysis

The audio-recorded interview sessions were transcribed ad verbatim in English by the interviewer (MIK). Data credibility or trustworthiness was assessed through respondent validation by giving the participants a chance to read the transcripts and evaluate if their feelings were represented in a fair manner [16]. None of the participants requested changes. All interview transcripts and analysis were organised using ATLAS.ti 8, a qualitative data analysis and research software [17]. The phenomenological methodology approach was chosen for this study [18]. It is a well-established approach that helps in elaborating a phenomenon by explaining and identifying the importance of people’s experiences [19]. The data were analysed using thematic analysis by Braun and Clarke [20], a method that is extensively recognized to be compatible with the phenomenological approach.

The method has a six-step process: familiarisation, coding, generating themes, reviewing themes, defining and naming themes, and writing up [20]. The first step, familiarisation, was done by the interviewer (MIK) by reading through the transcripts several times. Next, the transcripts were coded by highlighting sections of the text (phrases or sentences) and generation of shorthand labels or codes to describe the content. The initial coding was developed by MIK, and the transcripts were coded by MIK and SIK. Both MIK and SIK later generated relevant themes from the codes. The initial coding and broad-level themes derived from the data were reviewed by the supervisory team (MK, ZK, ZUH, NS) to make sure that the themes were useful and accurately represented the content. Each theme was defined and named, and the analysis was reported. The research team held regular meetings to discuss data collection, data quality, and analysis. To ensure that the qualitative methods were appropriate for the inquiry, the study was conducted and reported in accordance with the consolidated criteria for reporting qualitative research (COREQ) [21].

### 2.4. Ethics

Ethical approval to conduct the study was granted by the Human Research Ethics Committee of Universiti Sains Malaysia (USM/JEPeM/20010013) and the Khyber College of Dentistry, Department of Maxillofacial Surgery Peshawar, Khyber Pakhtunkhwa, Pakistan (Reference: OMFS/020/15).

## 3. Results

Characteristics of the individual participants are shown in Table 1. Most of the participants were male (76.5%). All were married. Their mean age was 50.9 years (SD 15.52) with the youngest being 25 and the oldest being 80. Most (70.6%) had received primary education and the remaining only up to secondary education. All participants were diagnosed with oral squamous cell carcinoma (OSCC). Most participants were at stage II (47.1%), followed by stage III (35.3%) and stage IV (17.6%).

We conducted 17 semi-structured interviews; one interview with each participant. All participants opted to have the interview at the Khyber College of Dentistry dental clinic. Eight major broad level themes were derived from the data: (1) pain and generalised physical weakness, (2) shock at diagnosis, (3) psychological distress of the COVID-19 pandemic, (4) faith and religion, (5) double hit loss of employment, (6) social isolation, (7) social support from caregivers, and (8) lack of support from health care professionals. 

### 3.1. Theme 1: Pain and Generalised Physical Weakness

The physical experiences of the participants at diagnosis were pain related to the condition. All participants reported pain located in the oral cavity which incapacitated them. The pain was found in three contexts: pain in the mouth, pain while eating and swallowing, unable to sleep because of pain.

Pain in the mouth and pain when eating and swallowing were commonly reported and variously expressed:

“*…I am in a lot of difficulty and pain. The pain has become so intolerable that I scream and shout and look at the sky…*”

(ID1)

“*I did not understand myself, if at all it is cancer it is only inside and not on the outside. Its only here on my tongue. That’s what it is, there is pain that starts from this end. I really don’t know what’s going on with me*.”

(ID10)

“*…I feel like my entire mouth is in pain. I cannot swallow at all...*”

(ID3)

“*Eating is completely compromised because my mouth and throat do not work, so how can I eat?*”

(ID17)

Almost all participants experienced sleep difficulties since their oral cancer diagnosis, and some of the participants pondered the origin of the issue to be associated with pain. One of the participants explained about disruption of sleep caused by oral pain:

“*Yes, I can’t sleep all night long. I am awake almost every night. I have sleepless nights because of the pain. How can I sleep?*”

(ID2)

Generalised physical weakness was commonly reported, and it was one of the important reasons cited for loss of spark and energy. The altered physical condition of weakness influenced them to live a passive lifestyle.

“*I do not move or walk around much and I do not go out. I prefer to stay home and be homebound. I stay home all the time and I no longer have the drive to go out like I used too*.”

(ID2)

The physical weakness led to consequences on their physical activity, and simple movement was experienced as a strenuous action. The experience of tiredness, loss of strength, and debility was the greatest barrier because it hindered participants from their normal daily life activities.

“…*I can’t carry out tasks of daily routine because I don’t possess much energy to perform everyday jobs*…”

(ID3)

“…*When I move around my head spins. Or like if I go from here to a few meters away, I wish to rest for a while*…”

(ID5)

Furthermore, some participants clarified that the physical inability and weakness were the fundamental reasons for their immobility, and dependency on others to help with their daily life activities.

“…*My lifestyle and physical health in the past twenty days has been that I am lying down in bed, completely flat on back*...”

(ID1)

“*I feel so weak that people have to help me while walking to the toilet. I feel really weak. I can’t really walk on my own.*”

(ID12)

### 3.2. Theme 2: Shock at Diagnosis

The psychological experiences of all participants began with shock at the time of diagnosis, particularly after receiving the definitive biopsy results confirming the diagnosis of oral cancer. The psychological struggles of receiving the bad news were so profound that the participants required efforts to understand and assimilate the diagnosis. Participants reported that at the time of diagnosis they felt shocked and anxious, and that deeply worried them.

“…*When I heard the news that I got very upset and was in a state of shock. I felt intensely disturbed, and I was hurt beyond my imagination*…”

(ID17)

“*The obvious thing is that when a person hears something like this; the shock, anxiety, and worries always kick in first*.”

(ID13)

The participants frequently linked the shock of diagnosis with the fear of the disease as a death sentence or a disease with an unknown outcome. A participant specified:

“…*At that point, I felt a huge burden and was shocked. I told myself these will be the last days of your life. I told myself you have been diagnosed and this is no less than losing life*…”

(ID16)

Nevertheless, the shock of the diagnosis also led to the determination to get through and survive. Many participants made attempts to seek and commence treatment. Some participants described discussing access to treatment information following oral cancer diagnosis.

“…*After diagnosis, the shock, and trauma, I had no choice but to seek treatment and visit this doctor to get further help*…”

(ID1)

“…*Psychologically I felt completely shocked when I was diagnosed with oral cancer. Besides I did not know where to get actual treatment from*…”

(1D12)

### 3.3. Theme 3: The Psychological Distress of the COVID-19 Pandemic

Almost all participants reported psychological distress related to the COVID-19 pandemic. Delays and restrictions imposed by the COVID-19 pandemic in accessing health care systems were the top concerns for most of the participants. Participants were more concerned about the postponement of their oral cancer treatment rather than contracting COVID-19. Participants explained increased stress levels and feeling hopeless:

“…*There are no doctors because the entire country is under lockdown. So, I am helpless, confused and bearing with all these problems without a ray of hope in such times*…”

(ID3)

“…*Added stresses were in store for me, when I arrived, I was informed everything has changed life, has come to a standstill due to the COVID-19 pandemic*…”

(ID9)

“*The hospitals are closed due to COVID-19. They keep saying there is corona, corona, corona. Corona has taken control of everything with itself and has closed the services down in hospitals for other treatments. When the hospitals are closed where do patients like us go?*”

(ID17)

The participants specified that besides postponement of treatment due to the COVID-19 pandemic, they were also concerned about the lack of hospital infrastructure including the medical personnel. The interrupted access to treatment affected them psychologically.

“…*I got the remaining three tests done like blood test and MRI. When I took those to the doctor, he said we don’t have space anymore in the hospital due to COVID-19*…”

(ID13)

“…*The biggest reason why I could not start getting my treatment is that all the public hospitals are closed due to COVID-19. I have been to the hospital three times, and they kept saying we can’t do your surgery because COVID-19 spread is immense, there is a pandemic emergency, and the hospitals don’t have enough vacant beds*…”

(ID15)

“…*Because of COVID-19 pandemic the hospitals are closed and not many doctors are available*...”

(ID16)

The psychological suffering of the participants also came from the additional financial challenges with the escalation of costs during the COVID-19 pandemic.

“…*Firstly, I have to come from so far away, whereas prices are increasing due to the pandemic*…”

(ID3)

“…*The doctor said that the rates will increase because of the COVID-19 issue. They said in the future the rates of getting treatment will differ*…”

(ID4)

“…*However, nowadays with the COVID-19 pandemic there is added stress and worries on me for example the situation is worse now. The transport charges are sky high requiring eight thousand or nine thousand for each travel to the city*…”

(ID7)

“…*These days the situation is bad because of COVID-19 and also everything has become so expensive*…”

(ID17)

### 3.4. Theme 4: Faith and Religion

All participants expressed that their faith and religion were the sources of psychological support and relief while being diagnosed with oral cancer during the COVID-19 pandemic. Most of the participants described having sturdy confidence in their religious belief. Many participants believed that they accepted the diagnosis of oral cancer because the disease was brought upon them by the will of God. Participants described their diagnosis of oral cancer with acceptance, in the context of their faith in God:

“*I thanked God for this disease. Because all diseases are from God, nowhere else*.”

(ID9)

“*Sickness comes from God side and only God. Nobody else has control over it, I cannot do anything or say anything else. God has brought it upon me, and God can take it away from me*.”

(ID10)

The participants also believed that their expectations of getting healed by treatment alone was not possible without the mercy of God. Most participants stated that while dealing with the diagnosis of oral cancer and looking for treatment, their inner faith and belief was of great assistance which provided them hope for betterment.

“*In all my health problems I ask God to make me better. Oh God make me better, Oh God put an end to my problems, Oh God I am thankful with all your decisions*.”

(ID9)

“*I say to myself that God is the best healer but there are various sources of help like doctors. It is their job, isn’t it? I believe God is the best healer and if doctors give me good treatment I will get better*.”

(ID12)

“…*These medical treatments are all blessings of good doctors and divine blessings of God all mighty*…”

(ID14)

The common forms of worship and connection with God among the participants were praying, crying, and recitation of the Holy Quran. There were regarded as religious sources of support:

“…*I will recite the Holy Quran and Pray because death is certain, but it is not certain when will a person die*…”

(ID10)

“…*Whenever I feel certain things, I wake up in the middle of the night and pray. I speak to God all mighty, I cry to Him, and he is the sole channel of communication*…”

(ID16)

### 3.5. Theme 5: Double-Hit Loss of Employment

In the opinion of most participants, loss of employment was a consequence of the oral cancer diagnosis. However, loss of employment was found in two contexts: loss of employment due to their physical inability and loss of employment-related to the COVID-19 pandemic.

Many participants believed that it would be difficult to keep a job due to functional limitation and disability related to oral cancer and its treatment. Some of the participants described how their altered physical condition had influenced their employment:

“…*After diagnosis, I haven’t been to work because I have been physically unable to do so*…”

(ID7)

“…*Yes, I was employed previously but now I am unemployed. I can’t keep a job because of my physical limitations and my state*…”

(ID16)

The COVID-19 pandemic affected job opportunities for some of the participants. The participants felt that the pandemic had forced unemployment to an unprecedented level. It was, therefore, sensible for them not to look for a job in the already overburdened socio-economic environment:

“…*On top of everything the country is going through bad employment circumstances due to COVID-19. I am sitting home and doing nothing*…”

(ID4)

“…*I used to work with an electric saw machine. Now I have lost my job. Ever since COVID-19 pandemic started*…”

(ID13)

Besides their own employment issues, some of the participants were worried about their family members, who have also lost their jobs or become unemployed during the COVID-19 pandemic. Participants expressed similar concerns about the changes in job market following the COVID-19 pandemic, that have affected the job security of their loved ones and putting more burden on them:

“…*One of my sons has just graduated recently but this COVID-19 pandemic has come. Unfortunately, he is unable to find a job, I was hoping he would get employed and be able to bear my treatment expenses*…”

(ID8)

“…*I have two sons one of them works and the other one doesn’t have a job because of the crippled job market these days during corona times. I’m worried sometimes we got an opportunity but, in these times, we don’t have anything*…”

(ID15)

### 3.6. Theme 6: Social Isolation

Most of the participants experienced social isolation which was reported in two contexts: decreased socialising due to their physical inability and reduced social interactions due to the COVID-19 distancing recommendation. Many participants reported that their ability to socialise was compromised after the diagnosis of oral cancer. The physical inability due to weakness associated with the disease hindered them from being socially active:

“…*No, socialising is not the same now just because I am weak now*…”

(ID5)

“…*Now I can’t visit my relatives, most of the times I stay in my room, and I can’t go outside. Before this I used to socialise and visit my relatives and used to do my own daytime work but now, I am not able to walk*…”

(ID14)

Many participants had been staying in bed all or most of the time due to the weakness. Restricting their physical activities by staying in bed disconnected them from their social life, leading to social withdrawal and isolation:

“*It has been two weeks since I have been on bed rest. Before that, I used to go out quite frequently*.”

(ID1)

“…*Now, since I am diagnosed with oral cancer, for a long time I have been on bed rest*…”

(ID7)

“…*I can’t go out and socialise anymore. I have spent this one month entirely on the bed. I really couldn’t walk*…”

(ID12)

Some participants also felt that their desire to meet and socialise was influenced by COVID-19 restrictions and social distancing measures. The participants described how they adjusted their social lives to avoid the infection and to comply with the social distancing regulations:

“…*Yes, I used to socialise quite often before but now due to COVID-19, I don’t wish to do so*...”

(ID3)

“…*Ever since I have been diagnosed with oral cancer, I no longer have friends rather stay at a distance from them because I have cancer and can’t afford any more problems*…”

(ID15)

### 3.7. Theme 7: Social Support from Caregivers

All participants shared experiences of receiving social support from their caregivers after being diagnosed with oral cancer. The caregivers were either immediate or close family members. Most participants appreciated the care and support they received and expressed satisfaction with the quality of care. In this regard, they stated:

“*All my daughter in laws are my caregivers and my sons also look after me*.”

(ID1)

“…*I have a beautiful family. May God give everyone a wife who is supportive like mine. Actually, a beautiful family and wife, both*…”

(ID5)

“…*And to be honest, whatever care is called I am getting it at home. I couldn’t have asked for more*…”

(ID10)

Most of the participants highlighted the important roles of the caregivers, particularly to help with the daily life activities such as eating, drinking, bathing, toileting, and dressing. The care and support that they received made them feel blessed:

“…*She looks after my medication time, and she asks me father what do you feel like eating? She really looks after all my needs*…”

(ID4)

“…*The love and care of mother, wife and daughter is way too different; these three relationships are a very big and a divine blessing. A wife can take you to the bathroom and can clean you up a mother can do the same, but no one can ever replace these relations*…”

(ID14)

“…*My husband looks after me and takes care of my needs. He gives me water to drink and makes an earning to put food on the table*…”

(ID17)

In addition to helping with the day-to-day activities, the caregivers also accompanied them during their visit to the hospital. This additional supportive role was expressed by the participants:

“*Yes, my youngest son is always with me. He is here with me today for my doctor’s appointment. He looks after me most of the time. Thanks to God Almighty*.”

(ID1)

“*My wife takes care of me and only her, she is my caregiver. She is my attendant while I am in the hospital, she’s here today*.”

(ID6)

“…*He has come with me to the hospital today. When I have his company during my hospital visits or elsewhere, I feel a little satisfied and feel happy*…”

(ID7)

### 3.8. Theme 8: Lack of Support from Health Care Professionals

All participants expressed the lack of support from the health care professionals. A few participants expressed their concerns about not knowing the hospital care team members whom they can contact to get the necessary information and care. In this aspect, they explained:

“…*There is no health care professional that is willing to give additional support in these times. I had to run around for myself*…”

(ID4)

“…*I have received no additional support from the health professionals in fact I have lost one lakh rupees trying to look for the right doctor. Various doctors kept on giving me various medications telling me I will get better with them but nothing*…”

(ID12)

“…*They don’t consider helping others in terms of seeking health care navigation, treatment, or other financial assistance*…”

(ID16)

Participants who visited the primary care settings also reported receiving little or no help from the health care professionals. In addition, there was no proper mechanism for referral of oral cancer patients to specialist cancer care treatment. In this respect, they stated:

“…*In my village there are mostly primary care staff who treat everything. There are no famous doctors and there is a hospital but not very functional since there aren’t many doctors available there*...”

(ID7)

“…*For the first time I consulted a primary care doctor in my own area, didn’t get much help*. …”

(ID13)

“…*The primary care doctors in the village can only give management by prescribing only medication and injection. The rest they say goodbye sit at home*…”

(ID17)

## 4. Discussion

The COVID-19 pandemic has significantly affected the overburdened health care system in Pakistan [22]. When the first wave of the pandemic hit the country, priorities of the health system were shifted [7]. Hospitals and health facilities were overwhelmed with COVID-19 patients, making it difficult for other patients with acute or chronic illness including oral cancer to access standard care [8]. The treatment outcomes of cancer are dependent on timely and high-quality multidisciplinary interventions [23]. This study has shown that the COVID-19 pandemic has clear impact on the life experiences of newly diagnosed oral cancer patients. The findings of this study demonstrated the classic and typical experiences of oral cancer diagnosis, with heightened psychosocial issues such as inability to access health care, psychological distress, and lack of social support from health care professionals during the pandemic.

The highly prevalent physical symptom at diagnosis of oral cancer is pain in the oral cavity which frequently co-exists with generalised physical weakness or fatigue leading to psychological distress [24,25]. The findings of our study on newly diagnosed oral cancer patients during the first wave of the COVID-19 pandemic are consistent with findings of pre-COVID-19 studies. The high levels of fatigue and pain among cancer patients during the COVID-19 pandemic has also been shown to increase the risk of mental health problems [26]. The psychological experiences among participants of this study began with the shock at diagnosis. The shock at diagnosis, often accompanied with significant reactions of disbelief and numbness, is a well-established experience among patients with head and neck cancer [27].

The psychological distress of COVID-19 identified in our study, however, revealed a diverse and seemingly contradictory psychological experience that has not been previously reported. The increased psychological distress during the COVID-19 pandemic was associated with issues such as disruption in treatment, inadequate health care infrastructure and personnel, and additional financial burdens with the upsurge in costs. The findings of our study are consistent with findings of a recent exploratory qualitative study that described the dilemma of cancer patients in accessing treatment whilst facing treatment delays or interruptions due to restricted access to health care services during the COVID-19 pandemic [28].

The treatment delays due to the COVID-19 pandemic is a dominant factor associated with increased levels of anxiety, fear, worry, and depression among diagnosed cancer patients [29,30]. The negative impact of the COVID-19 pandemic on patients was foreseeable with greater consequences in low-to-middle-income countries, due to having limited resources, obsolete infrastructure, shortage of health care providers, and a lack of ability to deliver care [31]. In agreement, the scarcity of health care personnel and inability of the health care system to provide optimal care and treatment were narrated by our participants. Our findings highlight some new insights into the heightened psychological adversity faced by oral cancer patients with increased financial burdens due to price hikes, which should be analysed in future research.

Evidence has suggested that religion and spirituality are associated with the physical health of cancer patients; whereby greater religion/spirituality is associated with better perceived physical health [32]. The psychological experience related to the philosophical doctrine of faith and religion is another salient finding from our study. All participants in this study were Muslims. Belief in destiny and fate is one of the basic beliefs of Islam [33]. The fundamental Islamic view that life and death are given by God explains the acceptance of the disease by most participants through their faith and religion after dealing with the shock of the diagnosis. Further qualitative and quantitative exploration on the influence of religious belief and faith on disease acceptance in other population groups is recommended. 

Social isolation faced by oral cancer patients from our study are consistent with a well-established fact, that patients diagnosed with oral cancer face social isolation due to their diminished physical ability to perform normal social activities [11]. Similarly, during the COVID-19 pandemic, the experience of loneliness and social isolation due to the COVID-19 restrictions could significantly decrease cancer patients’ quality of life were reported [34]. On the other hand, the social restrictions during the COVID-19 pandemic may not be entirely burdening for some; instead it resulted in a sense of social connectedness when everyone is advised to stay at home [34]. In agreement, some participants of our study expressed positive sentiments regarding social isolation and self-protection.

While the COVID-19 travel restrictions tend to reduce cancer patients social support due to lack of communication with their relatives and friends [35], findings from our study suggested differently. All participants reported receiving unwavering care and support from their caregivers. The social support experienced by our participants may be related to the traditional Pakistani culture that promotes social cohesion and interdependence, with the family forming the focal point of this strong social structure and support system [36,37]. The social support network for an individual is therefore extensive and includes the nuclear family, immediate relatives, distant relatives, tribe members, neighbours, and friends. This is different than the Western culture wherein social support is mostly provided by the nuclear family with little or no involvement of the extended family. Nevertheless, the strong family network and cohesion may lead to physical meetings and gatherings to care for the loved ones and must be weighed against the need to reduce the likelihood of infection transmission by adhering to the social restriction regulations. COVID-19 knowledge, attitude, and preventive practices have been shown to be low among the majority of the Pakistani population [38].

Participants in this study openly expressed the lack of support from health care professionals. These findings are congruent with other studies from Pakistan that also highlighted the issues of dysfunctional referral pathways for patients from the primary health care facilities such that patients tend to directly visit secondary and tertiary care hospitals [39,40]. Patient navigation and strong referral linkages have been shown to reduce barriers to cancer care in low-to-middle-income countries [41]. Strategies to ensure that patients receive the help they need to navigate their pathway to care is therefore essential for improving the outcomes of oral cancer patients in Pakistan.

Several qualitative studies have reported the impact of COVID-19 on cancer care, but the studies were mainly on patients with cancers found in sites other than the oral cavity [42,43,44]. Qualitative studies that explore the lived experiences of newly diagnosed oral cancer patients during the COVID-19 pandemic are scarce. To the best of our knowledge, this is the first qualitative study conducted with newly diagnosed oral cancer patients during the first wave of the COVID-19 pandemic in a low-to-middle-income country. The strength of qualitative research is that it can provide rich insights into the lived experiences that cannot be obtained by quantitative methods. However, while this study aimed to recruit a heterogeneous sample of newly diagnosed oral cancer patients by utilising the maximum variation purposive sampling method, the sampling strategy is limited by late presentation, where more than half of cases were diagnosed at a late stage (stage III and stage IV), and none were diagnosed at stage I. This issue of late presentation in oral cancer has been well-reported in past epidemiological studies [45]. 

## 5. Conclusions

The COVID-19 pandemic has a clear impact on the life experiences of newly diagnosed oral cancer patients. The cross-sectional results of this longitudinal qualitative study demonstrated the complexities and challenges of being diagnosed with oral cancer during the first wave of the COVID-19 pandemic. The themes identified in this study provide insight into the impact of the COVID-19 pandemic on the patient’s well-being. Our findings have important implications for strategies aimed at improving the physical, psychological, and social issues related to newly diagnosed oral cancer patients. 

Our findings also underline the need for effective patient-centred care to ensure continuity of accessible health care services for oral cancer patients during the COVID-19 pandemic. There have been several recommendations addressing the management of cancer patients during the COVID-19 pandemic [46,47,48]. These recommendations and findings of this study may help the government of Pakistan to assess and improve the quality of health services, which is relevant, not only in the current COVID-19 pandemic, but also future pandemics that are likely to be inevitable [49]. Efforts should also be made to develop an effective support program that incorporates appropriate and acceptable mental health services, and social support interventions, in response to the pandemic for newly diagnosed oral cancer patients in low-to-middle-income countries like Pakistan.

## Figures and Tables

**Table 1:** Demographic characteristics of participants.

Unique Identification (ID) Code	Age (Years)	Sex	Marital Status	Level of Education	Religion	Type of Cancer	Stage of Cancer
1	60	Male	Married	Secondary	Islam	OSCC	3
2	50	Female	Married	Primary	Islam	OSCC	2
3	70	Female	Married	Primary	Islam	OSCC	3
4	65	Male	Married	Primary	Islam	OSCC	2
5	60	Male	Married	Secondary	Islam	OSCC	2
6	45	Male	Married	Primary	Islam	OSCC	4
7	30	Male	Married	Secondary	Islam	OSCC	2
8	70	Male	Married	Primary	Islam	OSCC	3
9	45	Female	Married	Primary	Islam	OSCC	2
10	58	Male	Married	Secondary	Islam	OSCC	4
11	48	Male	Married	Secondary	Islam	OSCC	3
12	50	Male	Married	Primary	Islam	OSCC	3
13	40	Male	Married	Primary	Islam	OSCC	3
14	80	Male	Married	Primary	Islam	OSCC	4
15	25	Male	Married	Primary	Islam	OSCC	2
16	30	Male	Married	Primary	Islam	OSCC	2
17	40	Female	Married	Primary	Islam	OSCC	2

Abbreviation: OSCC, Oral squamous cell carcinoma.

## Data Availability

The data presented in this study are available on request from the corresponding author. The data are not publicly available due to restrictions set by the human research ethics committee.

## References

[1] World Health Organization (2011). ICD-10: International Statistical Classification of Diseases and Related Health Problems 10th Revision.

[2] Rivera C. (2015). Essentials of oral cancer. Int. J Clin. Exp. Pathol..

[3] Ferlay J., Soerjomataram I., Dikshit R., Eser S., Mathers C., Rebelo M., Parkin D.M., Forman D., Bray F. (2015). Cancer incidence and mortality worldwide: Sources, methods and major patterns in GLOBOCAN 2012. Int. J. Cancer.

[4] Bray F., Ferlay J., Soerjomataram I., Siegel R.L., Torre L.A., Jemal A. (2018). Global cancer statistics 2018: GLOBOCAN estimates of incidence and mortality worldwide for 36 cancers in 185 countries. CA Cancer J. Clin..

[5] Krishna Rao S.V., Mejia G., Roberts-Thomson K., Logan R. (2013). Epidemiology of oral cancer in Asia in the past decade--an update (2000–2012). Asian Pac. J. Cancer Prev..

[6] Malkani N., Kazmi S., Rashid M.U. (2021). Epidemiological assessment of oral cancer burden in Pakistan. Cancer Investig..

[7] Akhtar H., Afridi M., Akhtar S., Ahmad H., Ali S., Khalid S., Awan S.M., Jahangiri S., Khader Y.S. (2021). Pakistan’s response to COVID-19: Overcoming national and international hypes to fight the pandemic. JMIR Public Health Surveill..

[8] Saeed S., Tousif K., Fatir C.A., Basit J., Lee K.Y., Tahir M.J. (2022). Impact of COVID-19 on palliative care of cancer patients: Perspectives from Pakistan. Ann. Med. Surg..

[9] Neilson L., Kohli M., Munshi K.D., Peasah S.K., Henderson R., Passero V., Good C.B. (2022). Impact of the COVID-19 pandemic on new starts to oral oncology medications in the US. J. Oncol. Pharm. Pract..

[10] Lo Nigro C., Denaro N., Merlotti A., Merlano M. (2017). Head and neck cancer: Improving outcomes with a multidisciplinary approach. Cancer Manag. Res..

[11] Khattak M.I., Khan M., Khattak S.I., Khan Z., Haq Z.U., Saddki N. (2021). The experiences of oral cancer patients: A narrative review. Malays. J. Public Health Med..

[12] Schoonbeek R.C., Zwertbroek J., Plaat B.E.C., Takes R.P., Ridge J.A., Strojan P., Ferlito A., van Dijk B.A.C., Halmos G.B. (2021). Determinants of delay and association with outcome in head and neck cancer: A systematic review. Eur. J. Surg. Oncol..

[13] Forner D., Murnaghan S., Porter G., Mason R.J., Hong P., Taylor S.M., Bentley J., Hirsch G., Noel C.W., Rigby M.H. (2021). Psychosocial distress in adult patients awaiting cancer surgery during the COVID-19 pandemic. Curr. Oncol..

[14] Constantinou C.S., Georgiou M., Perdikogianni M. (2017). A comparative method for themes saturation (CoMeTS) in qualitative interviews. Qual. Res..

[15] Lincoln Y.S., Guba E.G. (1985). Naturalistic Inquiry.

[16] Birt L., Scott S., Cavers D., Campbell C., Walter F. (2016). Member checking: A tool to enhance trustworthiness or merely a nod to validation?. Qual. Health Res..

[17] Hwang S. (2008). Utilizing qualitative data analysis software: A review of Atlas. ti. Soc. Sci. Comput. Rev..

[18] Neubauer B.E., Witkop C.T., Varpio L. (2019). How phenomenology can help us learn from the experiences of others. Perspect. Med. Educ..

[19] Norlyk A., Harder I. (2010). What makes a phenomenological study phenomenological? An analysis of peer-reviewed empirical nursing studies. Qual. Health Res..

[20] Braun V., Clarke V., Cooper H., Camic P.M., Long D.L., Panter A.T., Rindskopf D., Sher K.J. (2012). Thematic analysis. APA Handbook of Research Methods in Psychology, Vol 2 Research Designs: Quantitative, Qualitative, Neuropsychological, and Biological.

[21] Tong A., Sainsbury P., Craig J. (2007). Consolidated criteria for reporting qualitative research (COREQ): A 32-item checklist for interviews and focus groups. Int. J. Qual. Health Care.

[22] Bhutta Z.A., Basnyat B., Saha S., Laxminarayan R. (2020). COVID-19 risks and response in South Asia. BMJ.

[23] Urruticoechea A., Alemany R., Balart J., Villanueva A., Viñals F., Capellá G. (2010). Recent advances in cancer therapy: An overview. Curr. Pharm. Des..

[24] Rigoni L., Bruhn R.F., De Cicco R., Kanda J.L., Matos L.L. (2016). Quality of life impairment in patients with head and neck cancer and their caregivers: A comparative study. Braz. J. Otorhinolaryngol..

[25] Kanchan S., Pushpanjali K., Tejaswini B.D. (2019). Challenges faced by patients undergoing radiotherapy for oral cancer: A qualitative study. Indian J Palliat. Care.

[26] Wang Y., Duan Z., Ma Z., Mao Y., Li X., Wilson A., Qin H., Ou J., Peng K., Zhou F. (2020). Epidemiology of mental health problems among patients with cancer during COVID-19 pandemic. Transl. Psychiatry.

[27] Lang H., France E., Williams B., Humphris G., Wells M. (2013). The psychological experience of living with head and neck cancer: A systematic review and meta-synthesis. Psychooncology.

[28] Colomer-Lahiguera S., Ribi K., Dunnack H.J., Cooley M.E., Hammer M.J., Miaskowski C., Eicher M. (2021). Experiences of people affected by cancer during the outbreak of the COVID-19 pandemic: An exploratory qualitative analysis of public online forums. Supportive Care Cancer.

[29] De Joode K., Dumoulin D.W., Engelen V., Bloemendal H.J., Verheij M., van Laarhoven H.W.M., Dingemans I.H., Dingemans A.C., van der Veldt A.A.M. (2020). Impact of the coronavirus disease 2019 pandemic on cancer treatment: The patients’ perspective. Eur. J. Cancer.

[30] Guven D.C., Sahin T.K., Aktepe O.H., Yildirim H.C., Aksoy S., Kilickap S. (2020). Perspectives, knowledge, and fears of cancer patients about COVID-19. Front. Oncol..

[31] Bong C.L., Brasher C., Chikumba E., McDougall R., Mellin-Olsen J., Enright A. (2020). The COVID-19 pandemic: Effects on low- and middle-income countries. Anesth. Analg..

[32] Jim H.S., Pustejovsky J.E., Park C.L., Danhauer S.C., Sherman A.C., Fitchett G., Merluzzi T.V., Munoz A.R., George L., Snyder M.A. (2015). Religion, spirituality, and physical health in cancer patients: A meta-analysis. Cancer.

[33] Elaskary M., Yun E.K. (2017). Death, resurrection, and shrine visitations: An Islamic perspective. Religions.

[34] Schellekens M.P.J., van der Lee M.L. (2020). Loneliness and belonging: Exploring experiences with the COVID-19 pandemic in psycho-oncology. Psychooncology.

[35] Liu X., Liu F., Tong F., Peng W., Wen M., Zou R., Zhang L., Jiang L., Yang H., Yi L. (2020). Psychological reactions and interventions to help cancer patients cope during the COVID-19 pandemic in China. J. Psychosoc. Oncol. Res. Pract..

[36] Banning M., Hafeez H., Faisal S., Hassan M., Zafar A. (2009). The impact of culture and sociological and psychological issues on Muslim patients with breast cancer in Pakistan. Cancer Nurs..

[37] Afzal A., Chuang S.S., Moodley R., Gielen U.P., Akram-Pall S. (2021). Pakistani families. Asian Families in Canada and the United States: Advances in Immigrant Family Research.

[38] Salman M., Mustafa Z.U., Asif N., Shehzadi N., Hussain K., Khan T.M., Mallhi T.H., Khan Y.H., Butt M.H., Farrukh M.J. (2021). Awareness of COVID-19 among illiterate population in Pakistan: A cross-sectional analysis. Disaster Med. Public Health Prep..

[39] Khalid F., Abbasi A.N. (2018). Challenges faced by Pakistani healthcare system: Clinician’s perspective. J. Coll. Physicians Surg. Pak..

[40] Aziz S.Z., Hanif I. (2016). Primary care and health system performance in Pakistan: A study of basic health units of South Punjab. J. Pak. Med. Assoc..

[41] Dalton M., Holzman E., Erwin E., Michelen S., Rositch A.F., Kumar S., Vanderpuye V., Yeates K., Liebermann E.J., Ginsburg O. (2019). Patient navigation services for cancer care in low-and middle-income countries: A scoping review. PLoS ONE.

[42] Lesley G.C., Tahmasebi H., Meti N., Wright F.C., Thawer A., Cheung M., Singh S. (2021). Cancer treatment during COVID-19: A qualitative analysis of patient-perceived risks and experiences with virtual care. J. Patient Exp..

[43] Drury A., Eicher M., Dowling M. (2021). Experiences of cancer care during COVID-19: Phase 1 results of a longitudinal qualitative study. Int. J. Nurs. Stud. Adv..

[44] Seven M., Bagcivan G., Pasalak S.I., Oz G., Aydin Y., Selcukbiricik F. (2021). Experiences of breast cancer survivors during the COVID-19 pandemic: A qualitative study. Supportive Care Cancer.

[45] Warnakulasuriya S. (2009). Global epidemiology of oral and oropharyngeal cancer. Oral Oncol..

[46] Jafari A., Rezaei-Tavirani M., Karami S., Yazdani M., Zali H., Jafari Z. (2020). Cancer care management during the COVID-19 pandemic. Risk Manag. Healthc..

[47] Curigliano G., Banerjee S., Cervantes A., Garassino M.C., Garrido P., Girard N., Haanen J., Jordan K., Lordick F., Machiels J.P. (2020). Managing cancer patients during the COVID-19 pandemic: An ESMO multidisciplinary expert consensus. Ann. Oncol..

[48] González-Montero J., Valenzuela G., Ahumada M., Barajas O., Villanueva L. (2020). Management of cancer patients during COVID-19 pandemic at developing countries. World J. Clin. Cases.

[49] Van Kerkhove M.D., Ryan M.J., Ghebreyesus T.A. (2021). Preparing for “Disease X”. Science.

